# RUNX1 and FOXP3 interplay regulates expression of breast cancer related genes

**DOI:** 10.18632/oncotarget.6771

**Published:** 2015-12-28

**Authors:** María Sol Recouvreux, Esteban Nicolás Grasso, Pablo Christian Echeverria, Luciana Rocha-Viegas, Lucio Hernán Castilla, Carolina Schere-Levy, Johanna Melisa Tocci, Edith Claudia Kordon, Natalia Rubinstein

**Affiliations:** ^1^ Instituto de Fisiología, Biología Molecular y Neurociencias (IFIBYNE-UBA-CONICET), Buenos Aires, Argentina; ^2^ Departamento de Fisiología y Biología Molecular y Celular, Facultad de Ciencias Exactas y Naturales, UBA, Buenos Aires, Argentina; ^3^ Departamento de Química Biológica, UBA, Buenos Aires, Argentina; ^4^ Department of Molecular, Cell and Cancer Biology, University of Massachusetts Medical School, Worcester, MA, USA; ^5^ Department of Biologie Cellulaire, Universite de Geneve Sciences III, Geneve, Switzerland; ^6^ Present Address: Oncology Institute “Angel H Roffo”, Buenos Aires, Argentina; ^7^ Present Address: Immunopharmacology Laboratory, IQUIBICEN-CONICET, FCEN-UBA, Buenos Aires, Argentina

**Keywords:** Runx1, Foxp3, Rspo3, GJA1, gene expression regulation

## Abstract

Runx1 participation in epithelial mammary cells is still under review. Emerging data indicates that Runx1 could be relevant for breast tumor promotion. However, to date no studies have specifically evaluated the functional contribution of Runx1 to control gene expression in mammary epithelial tumor cells. It has been described that Runx1 activity is defined by protein context interaction. Interestingly, Foxp3 is a breast tumor suppressor gene. Here we show that endogenous Runx1 and Foxp3 physically interact in normal mammary cells and this interaction blocks Runx1 transcriptional activity. Furthermore we demonstrate that Runx1 is able to bind to R-spondin 3 (RSPO3) and Gap Junction protein Alpha 1 (GJA1) promoters. This binding upregulates Rspo3 oncogene expression and downregulates GJA1 tumor suppressor gene expression in a Foxp3-dependent manner. Moreover, reduced Runx1 transcriptional activity decreases tumor cell migration properties. Collectively, these data provide evidence of a new mechanism for breast tumor gene expression regulation, in which Runx1 and Foxp3 physically interact to control mammary epithelial cell gene expression fate. Our work suggests for the first time that Runx1 could be involved in breast tumor progression depending on Foxp3 availability.

## INTRODUCTION

The RUNX proteins belong to a family of transcription factors (RUNX1, 2 and 3) known to play crucial roles in hematopoiesis, osteogenesis and neurogenesis [1]. RUNX1 activity has been comprehensively study in physiological and tumor contexts [2, 3]. It has a runt domain and is able to bind a common TG (T/C)GGT consensus binding site, inducing proliferation in a context-dependent manner [4–6]. Moreover, RUNX1 is considered as a multifaceted protein that associates with diverse partners to direct different biological outcomes [2]. In particular Runx1 has both transcriptional activation and inhibition domains that allow it to bind a plethora of co-factors, such as the tumor suppressor gene Foxp3, which in turn modulates Runx1's regulatory effect [7, 8]. Sakaguchi and colleagues showed that Foxp3 transcription factor inhibits Runx1 transcriptional activity by protein-protein interaction promoting the suppressive function of regulatory T cells [8]. In human breast cancer, Runx1 activity is still matter of debate and little is known about its direct role in breast cancer progression [9–12]. Interestingly, Ferrari and colleagues have shown using multivariate analysis that high expression of RUNX1 correlates with poor prognosis in triple negative human breast cancer and strongly suggest that Runx1 could be used as an independent prognostic marker in this subgroup of human breast cancer [13].

Foxp3 is an X-linked tumor suppressor gene expressed in the normal mammary gland, but downregulated, mutated or cytoplasmically localized in mammary tumor cells [14–18]. It has been shown that in normal cells, wild-type FOXP3 is bound to the promoter and transcriptionally repress human epidermal growth factor receptor Her-2/Neu and miR-146a promoters, transcriptionally repressing expression of these mediators that are involved in mammary tumorigenesis [15, 19]. Moreover, FOXP3 overexpression in human cancer cell lines was shown to repress tumor growth and metastasis [15, 19, 20].

This context encouraged us to investigate if Runx1 is able to modulate gene expression in mammary tumor cells, and whether this activity could be modulated by Foxp3 in normal epithelial cells. To achieve this goal, we investigated Runx1 and Foxp3 participation in the regulation of expression of two tumor associated genes, *Rspo3* [21, 22] and *GJA1* [23, 24], which are known modulators of breast tumor cell growth (positively and negatively, respectively). Both promoter regions possess Runx1 binding sites, but no Foxp3-binding regions were detected in their proximity. Runx1 is able to promote RSPO3 gene expression and inhibit GJA1 gene expression on tumor epithelial cells, depending on Foxp3 availability. Our results show, for the first time, that Foxp3 thwarts Runx1 activity through physical interaction in mammary epithelial cells. Furthermore, these data suggest that Runx1 might modulate mammary gland tumorigenesis depending on Foxp3 expression levels unraveling a new mechanism of gene expression regulation on mammary epithelial cells.

## RESULTS

### Runx1 activates RSPO3 oncogene expression in tumor cells

R-spondin protein 3 (RSPO3) belongs to a family of secreted proteins that strongly potentiates Wnt/βcatenin signaling [25, 26] and regulates tissue patterning and differentiation [27, 28]. In particular, RSPO3 has been described as a potent oncogene due to its ability to transform and generate mammary tumors *in vivo* after inoculation of RSPO3-transduced epithelial mammary cells [22]. Furthermore, we and other laboratories, described that MMTV-induced mammary gland tumors express high levels of RSPO3 compared with virgin normal mammary gland [21, 22]. To address the question of how this oncogene expression is differentially regulated in normal and tumor mammary epithelial cells, we analyze the promoter region of RSPO3. *In silico* analysis of *Rspo3* promoter region (1500 bp upstream from +1 transcription start site) revealed three putative binding sites for the transcription factor Runx1: two of high affinity (TG (T/C) GGT) and one of low affinity (AGTGGT) (Supplementary Table 1). While, no Foxp3 binding sites (A/GTAAACAA) were found.

**Figure 1:**
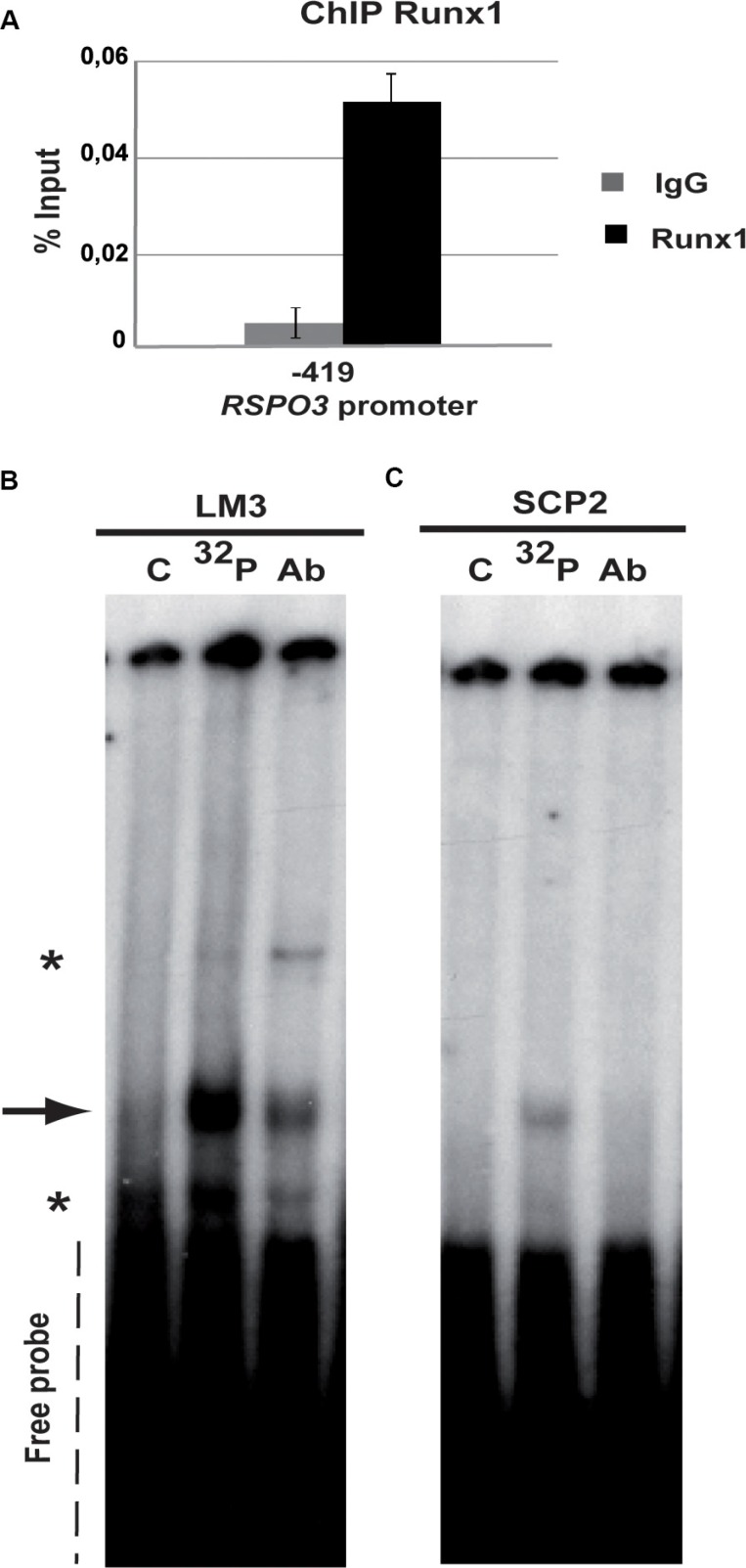
Runx1 binds to *Rspo3* promoter (**A**) ChIP assays were performed on LM3 cells using specific ChIP-grade Runx1 antibody or control IgG antibody. Specific primers were designed for targeting Runx1 high affinity binding site in the *Rspo3*'s promoter region. Bar graph shows mean and standard deviation (SD) of three independent experiments each of them performed by triplicate. Primers for *Gapdh* promoter region were used as negative control with no amplification product. (**B–C**) Gel shift assays were performed on LM3 (B) or SCp2 (C) nuclear extracts using an oligoprobe containing *Runx1* consensus sequence included in the *Rspo3* promoter region (−490bp) (lane ^32^P and lane Ab). This band showed lower intensity when cold oligonucleotides were included in the reaction (lane C). Asterisks in the Figure show unspecific binding. ^32^P: phospho-labeled oligoprobe, Ab: anti-Runx1 antibody and C unlabeled oligoprobe.

**Figure 2:**
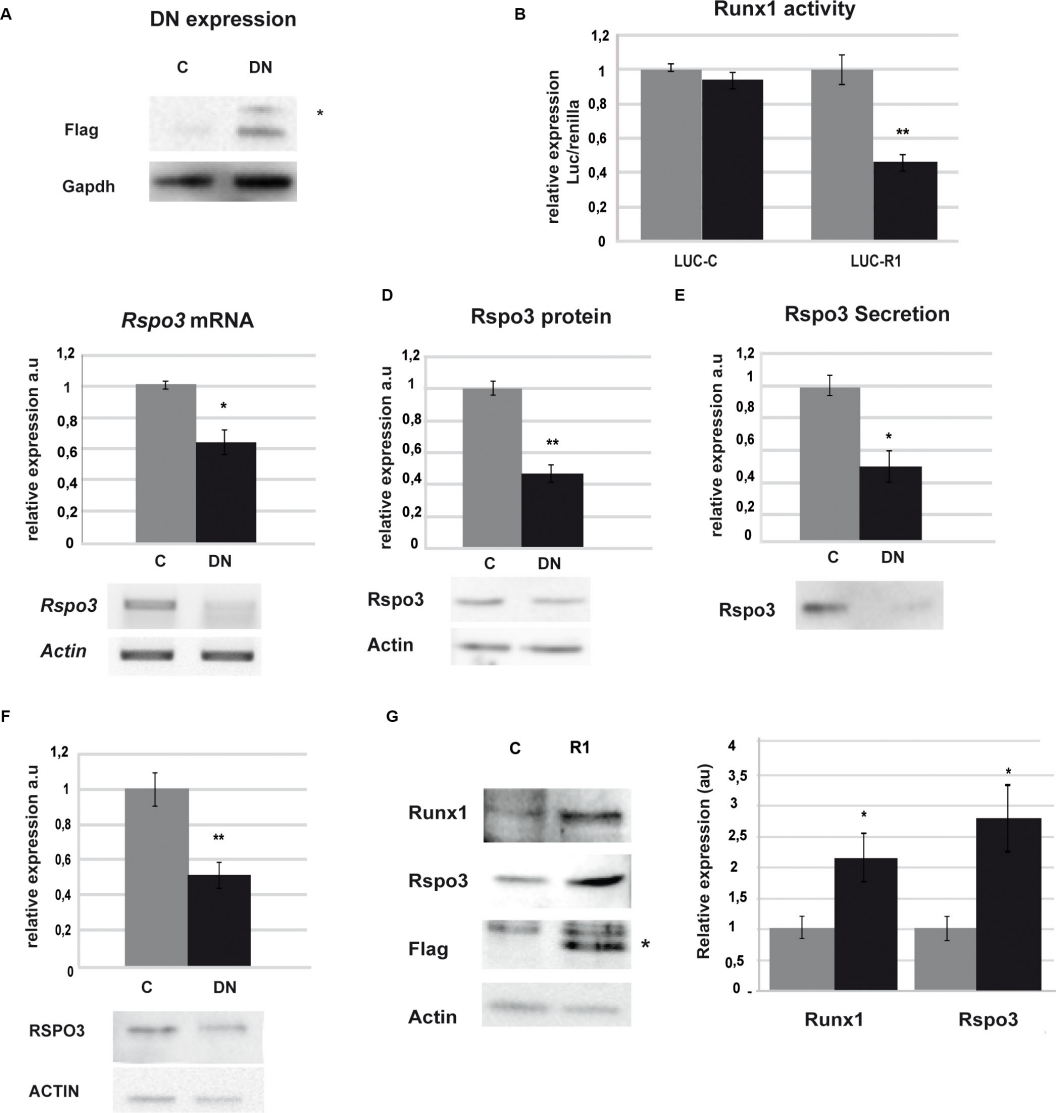
Runx1 regulates *Rspo3* expression (**A–F**) LM3 and MDA-MB-231 cells were transfected with DN or C. (A) Western blot (WB) showing tag flag expression in LM3 cells 48 h after transfection. Asterisk indicates specific band. (B) Luciferase assays on LM3 cells 48 h after co-transfection with DN or C and a reporter vector of Runx1 activity (Luc-R1) or empty vector, as control (Luc-C). Bar graph showing mean and SD of three independent experiments (*p* = 0,0052) (C–D) *Rspo3* mRNA levels by semi-quantitative RT-PCR (C) and protein by WB (D) on LM3 cells 48 h after transfection. Bar graph showing mean and SD of three independent experiments (*p* = 0.014 and (*p* = 0,007). (E) Rspo3 protein secretion levels by WB of conditioned media generated by LM3 cells 48 h after transfection. Values were normalized to the number of cells after transfection. Bar graph showing mean and SD of three experiments (*p* = 0.021). (F) RSPO3 protein levels by WB analysis on MDA-MB-231 48 h after transfection. Bar graph showing mean and SD of three independent experiments (*p* = 0.0047). (**G**) SCP2 cells were transfected with a vector containing full Runx1 cDNA (R1) or empty vector as control (C) and WB analysis of Runx1 (first line), Rspo3 (second line), Flag tag (third line) and *Actb* (fourth line, used as loading control) were performed. Asterisk indicates specific band. Bar graph shows WB quantification, C in grey columns and R1 in black columns. Bar graph showing mean and SD of three independent experiments (*p* = 0,042).

We then investigated the potential role of Runx1 in the regulation of Rspo3 gene expression, in the LM3 cell line, which was derived from a spontaneous BALB/c mouse mammary tumor [29]. LM3 cells can generate metastatic tumors when inoculated into syngeneic mice [30]. The LM3 cell line expresses detectable levels of Rspo3 mRNA (Supplementary Figure 1) and a transcriptionally active form of Runx1, which binds to the consensus sequence found in the Rspo3 promoter region (Figure 1A–1B and Figure 2B). In the gel shift assay the signal intensity decreases when cold oligonucleotide is included in the reaction (Figure 1B, 1C lane versus ^32^P lane) showing the specificity of the DNA-protein binding. Furthermore, when nuclear extracts were co-incubated with the labelled probe and an anti-Runx1 antibody, the intensity of the band decreased (Figure 1B, 1AB lane versus ^32^P lane), probably because the antibody interferes with Runx1 DNA binding domain. These results suggest that endogenous Runx1 is able to bind its putative binding site in the *rspo3* promoter.

To evaluate if the observed DNA/Runx1 interaction is biologically relevant for Rspo3 expression, we altered Runx1 expression levels in tumor and normal cell lines and evaluated Rspo3 expression and cell behaviour changes. Runx1 transcriptional activity was reduced by expression of the dominant-negative (DN) form of Runx1 in LM3 and MDA-DB-231 tumor cells [31]. We observed a significant reduction of Runx1 transcriptional activity in DN/Runx1 transfected tumor cells (Figure 2A and 2B), which resulted in a significant downregulation of Rspo3 expression and secretion (Figure 2C–2E: LM3 cell line and 2F: MDA-MB-231 cell line). On the other hand, we transfected SCp2 non-tumor epithelial mammary cells with an expression vector containing the full length cDNA sequence of *Runx1* down-stream of a CMV-promoter [32]. Figure 2G shows that overexpression of Runx1 in these cells induced significant upregulation of Rspo3 expression. These experiments demonstrate that Runx1 is able to bind to *Rspo3* promoter and triggers the expression of this oncogene in mammary epithelial cells.

### Runx1 and Foxp3 physically interact in normal mammary epithelial cells

**Figure 3:**
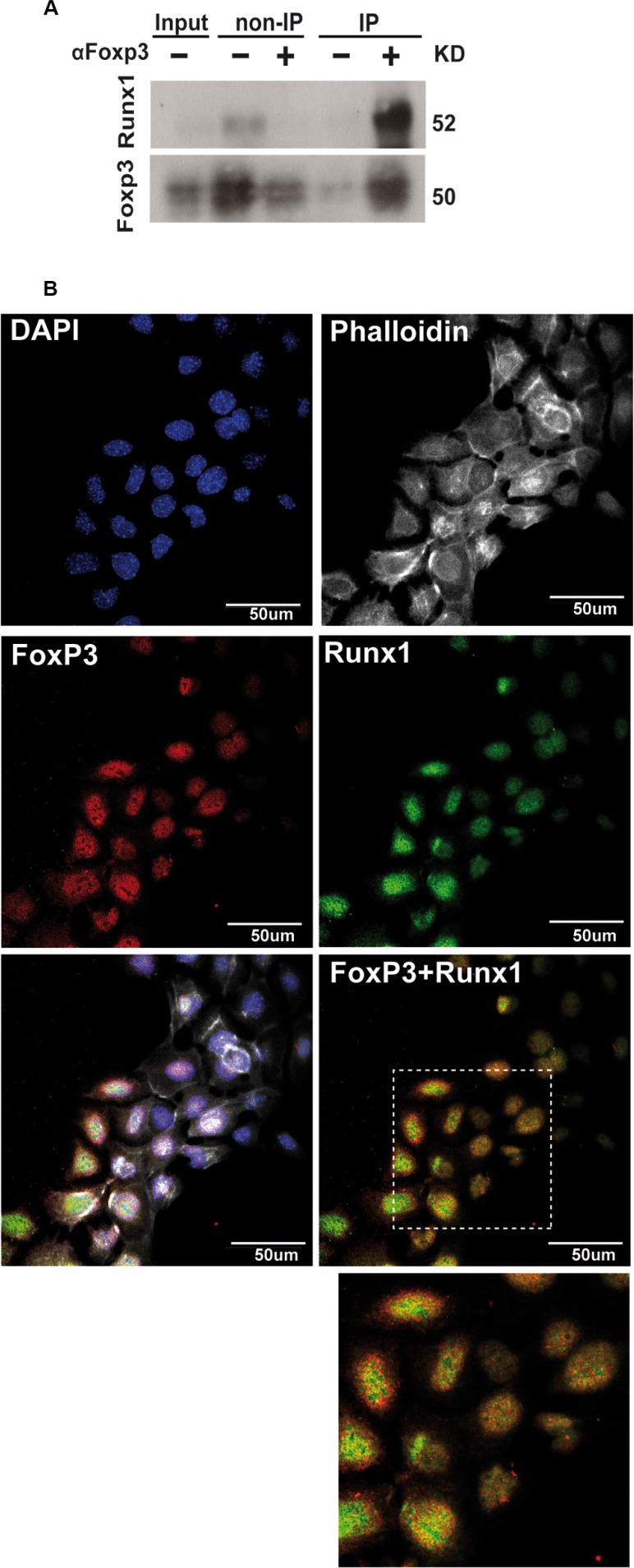
Runx1 physically interacts with Foxp3 in normal mammary epithelial cells (**A**) Total protein extracts from SCp2 cells were incubated with anti-Foxp3 antibody and subsequent precipitation products were analyzed with anti-Runx1 antibody by WB analysis. IP (+) lane shows Runx1 co-immunoprecipitated with Foxp3. (**B**) Immunofluorescence of SCp2 cells shows subcellular localization of Runx1 (green) and Foxp3 (red) by confocal microscopy. Merge and inset figures show yellow dots representing co-localization of Runx1 and Foxp3 proteins. Magnification bar: 50 μm.

It has been previously shown that Foxp3 can interact with Runx1 and block its transcriptional activity in regulatory T cells [8]. Normal mammary epithelial cells express higher functional levels of Foxp3 compared to tumor cells [15] and comparable levels of endogenous *Runx1* mRNA (Supplementary Figure 1A). To investigate if Foxp3 is able to modulate Runx1 transcriptional activity in normal mammary cells, we first explored whether endogenous Runx1 and Foxp3 proteins physically interact in these cells. To address this question we performed co-immunoprecipitation assays in which total protein extracts from SCp2 cells were incubated with anti-Foxp3 antibody and the precipitated proteins were immunoblotted with an anti-Runx1 antibody. Figure 3A shows a band of 50KDa corresponding to Runx1 molecular weight in the IP lane (co-immunoprecipitated proteins). Furthermore, Figure 3B shows that endogenous Runx1 and Foxp3 are concentrated in the nuclear compartments. In addition, partial co-localization of these two proteins in SCp2 cell nuclei is observed (Figure 3B). Therefore, these studies indicate that Runx1 and Foxp3 proteins physically interact in this mammary epithelial cell line.

### Foxp3 blocks Runx1 transcriptional activity in mammary epithelial cells

**Figure 4:**
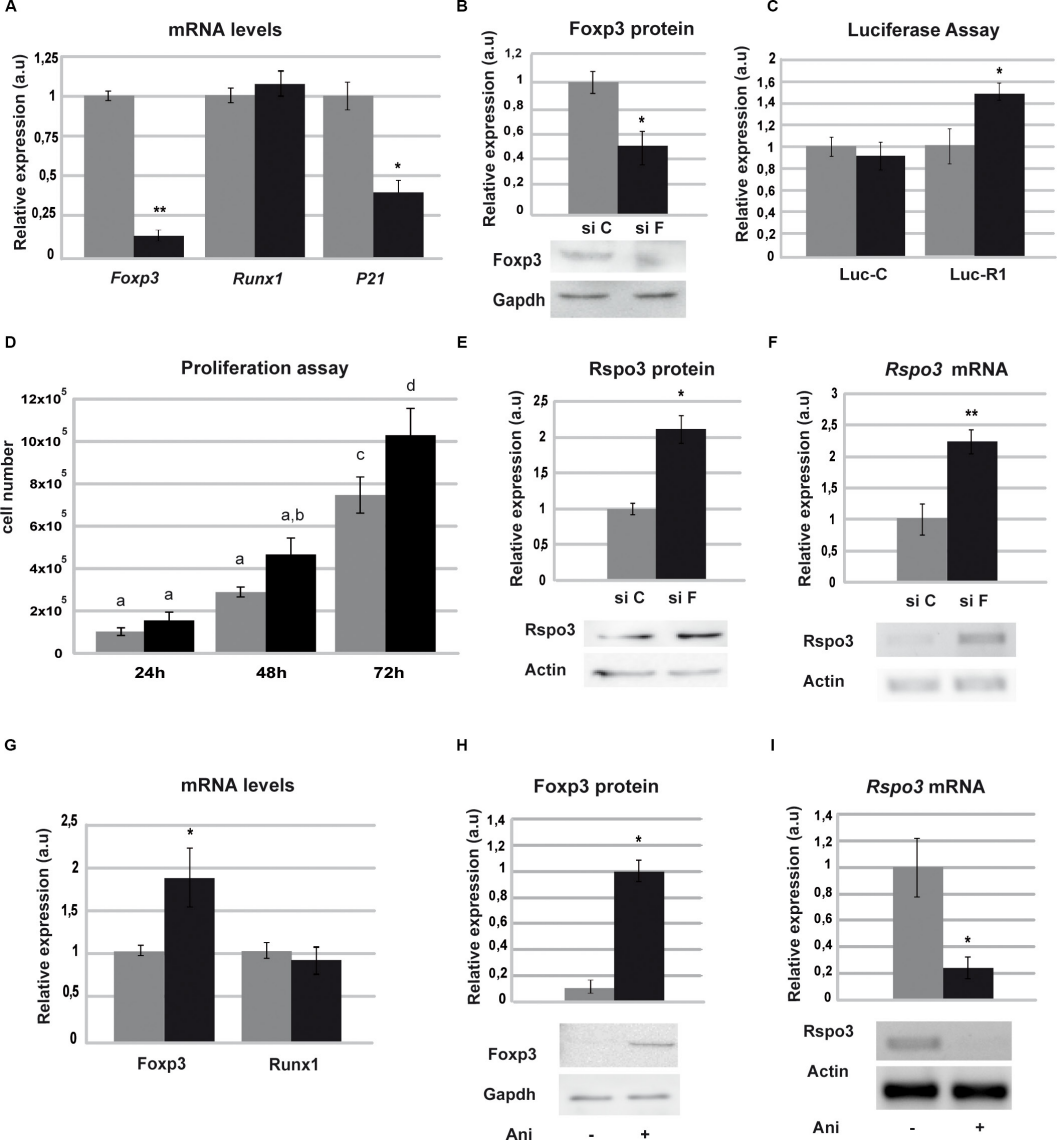
Foxp3 blocks Runx1 transcriptional activity SCp2 cells were transfected with specific Foxp3 siRNA (siF) or scrambled control (siC) and mRNA or total protein extracts were analyzed. (**A**) Downregulation of Foxp3 levels (*p* = 0.004) (left), Runx1 (middle) and *p21* (right) (*p* = 0.042) expression by RT-qPCR. (**B**) Downregulation of Foxp3 levels by WB (*p* = 0.013), Gapdh expression was used as loading control. (**C**) SCp2 cells were co-transfected with Luc-R1 or Luc-C and luciferase assay was performed (*p* = 0.028). (**D**) Proliferation assay was performed by counting cell number after siF and siC treatment (ANOVA p < 0.0001). Different letters means significant differences *p* < 0.05 by Bonferroni contrast. (**E–F**) *Rspo3* expression by WB (*p* = 0.016) (E) and by PCR (*p* = 0.0059) (F) upon siF or siC treatment. *Actd* expression was used as loading control. Images were analyzed with image J software. In each case histogram shows mean and SD of three independent experiments, siC in grey bars and siF in black bars. (**G–I**) LM3 cells were incubated with Anisomycin and total protein or RNA extracts were analyzed 2 h after treatment. (G) RT-qPCR of *Foxp3* (*p* = 0.04) (left) and *Runx1* (right) were normalized to *actin* levels in Anisomycin treated (black bars) and control (gray bars) cells. (H) WB of Foxp3 with (black bars) or without (gray bars) Anisomycin and Gapdh was used as loading control (*p* = 0.022). (I) *Rspo3* expression by RT-PCR. Histogram shows semi-quantification of the intensity of the band from three independent assays performed with vehicle (gray bars) or Anisomycin (black bars) (*p* = 0.012).

The SCp2 cell line showed visible mRNA levels of Foxp3, while Rspo3 endogenous expression was undetectable (Supplementary Figure 1A). Figure 1C also shows that in these cells, differently to what we have observed in LM3 cells, a lesser Runx1 binding to its consensus sequences. To study whether endogenous Foxp3 might be responsible for inhibition of Runx1 binding to the DNA, we evaluated Runx1 transcriptional activity after transfecting SCp2 cells with either Foxp3 (siF) or control (siC) siRNAs. Figure 4A and 4B show that Foxp3 is downregulated in SCp2 siF-transfected cells, while Runx1 expression levels are not modified (Figure 4A). Knockdown of Foxp3 in SCp2 cells led to a decrease in p21 expression (Figure 4A), a known Foxp3 target gene [33] and significantly prompted SCp2 proliferation, previously described *in vivo* in [33] (Figure 4D). Importantly, our results show that Foxp3 downregulation resulted in a significantly enhanced Runx1 transcriptional activity (Figure 4C) and Rspo3 expression induction (Figure 4E and 4F).

In addition, to determine whether induction of Foxp3 expression may result in inhibition of Runx1 target genes expression in mammary tumor cells (LM3 cells), which show low levels of Foxp3 (Supplementary Figure 1), were treated with low doses of anisomycin which is able to upregulate Foxp3 expression in mammary epithelial cell lines (Figure 4G) by c-Jun and ATF-2 activation [34]. Figure 4G–4I) shows that anisomycin treatment of LM3 cells caused a decrease in *Rspo3* expression (4H–4I) without affecting the levels of *Runx1* mRNA (4G).

Taken together, these experiments indicate that Foxp3 might be able to impair Runx1 transcriptional activity in normal mammary epithelial cells. In tumor cells Foxp3 low expression would explain high transcriptional activity of *Runx1* on *Rspo3* promoter. Furthermore, these data strongly suggest that Foxp3 and Runx1 protein interaction could determine Runx1 tumor promotion transcriptional activity in mammary epithelial cells. Moreover, our data propose a new tumor suppressor role for Foxp3 in mammary epithelial cells.

### Impaired Runx1 activity reduces migration of breast tumor cells and upregulates GJA1 gene expression

**Figure 5:**
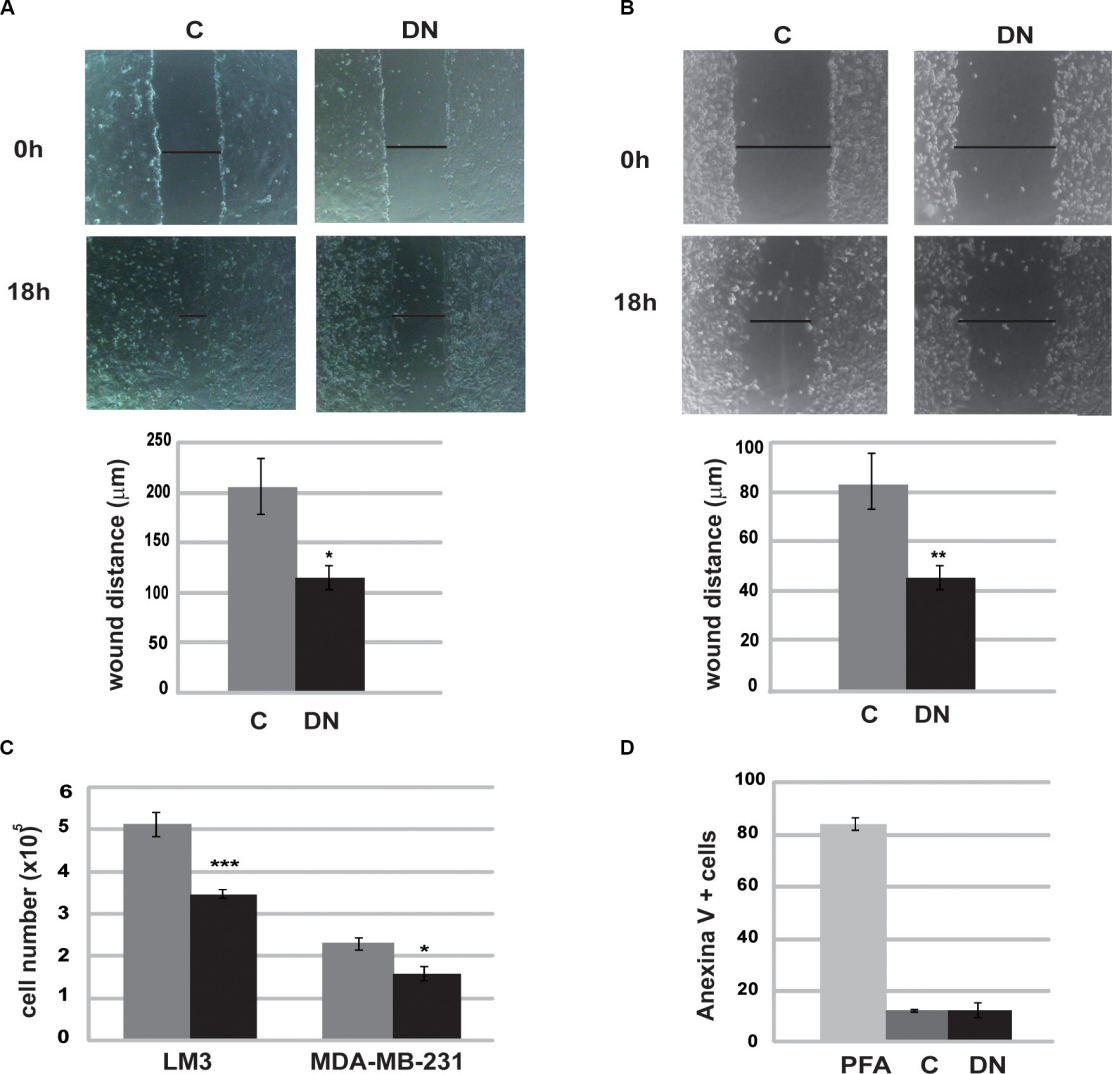
Runx1 is required for tumor cell migration LM3 cells (**A**) and MDA-MB-231 cells (**B**) were subjected to wound healing assays for 18 h after transfection with DN or C expression vectors. Time points: 0 h and 18 h. A representative assay (up) and histograms (down) show mean and SD of three independent experiments (LM3 *p* = 0.02; MDA *p* = 0,0035). (**C**) LM3 and MDA-MB-231 cell number were assessed before and after transfection with DN or C expression vectors. Histogram shows mean and SD of three independent experiments (LM3 *p* = 0.0007; MDA *p* = 0,03) Axis X is 10^5^ cells. (**D**) After transfection with DN or C expression vectors, LM3 cells were incubated with Annexin V-FITC and PI. Apoptosis was measure by flow citometry. PFA 4% was used as positive control.

As tumor metastasis is the main cause of morbidity and mortality, we explored the ability of Runx1 to promote tumor aggressiveness. To address this question we studied migration capacity of DN/Runx1 (DN) transfected tumor cells (LM3 and MDA-MB-231) by *in vitro* scrape-wound closure assays. Our results show that Runx1 inhibition resulted in a lower ability to fill the area by cell migration (Figure 5A and 5B). In addition, reduction in Runx1 transcriptional activity was also accompanied by a reduced number of cells (Figure 5C) but no changes in apoptosis levels (Figure 5D), indicating that Runx1 might be involved in cell cycle regulation without inducing cell death.

**Figure 6:**
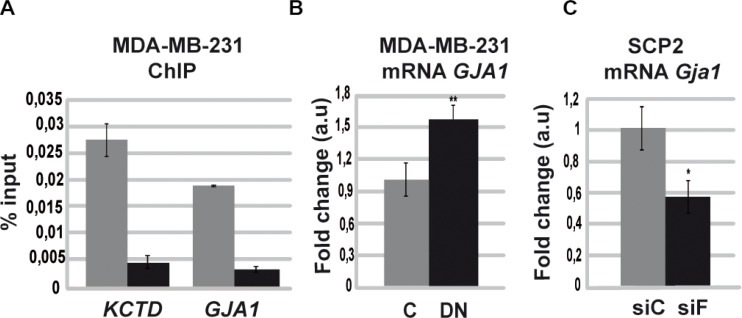
Runx1 downregulates GJA1 gene expression in the absence of Foxp3 (**A**) ChIP assays were performed on MDA-MB-231 cells using specific ChIP-grade RUNX1 antibody or control IgG. Primers targeting RUNX1 high affinity binding site included in the *GJA1*'s promoter region were designed. Histogram shows mean and SD of three independent experiments performed by triplicate. Primers for *GAPDH* promoter region were used as negative control with no amplification product. Primers for KCTD promoter region were used as positive control previously described in [58] (KCTD: Potassium Channel Tetramerization Domain). Anti-Runx1 antibody is in grey bars and Anti-IgG antibody is in black bars. (**B**) *GJA1* mRNA levels by qPCR of MDA-MB-231 transfected cells with DN or C expression vector. Histograms represent mean and SD of three independent experiments (*p* = 0.004). (**C**) SCp2 cells were transfected with specific Foxp3 siRNA (siF) or scrambled control (siC) and *GJA1* gene expression was measured by RT-qPCR. Histograms represent mean and SD of three independent experiments (*p* = 0.03).

Since Runx1 is a potent regulator of gene transcription in different cell types [1, 4, 5, 7, 35], we hypothesized that the effect observed when tumor cells expressed the DN/Runx1 might have a wider effect on genome expression of breast cancer cells. Therefore, we performed an *in silico* analysis that provided us a list of Runx1 probable target genes that were classified by their biological functions using Gene Ontology database. Only those previously described in breast cancer were selected (MS Recouvreux, unpublished data). This approach revealed that Runx1 is potentially able to regulate *Gja1* (Supplementary Table 1) among other genes (MS Recouvreux et al. unpublished data). Interestingly, GJA1 has been described as a tumor-suppressor gene, which is able to reduce migration and proliferation of MCF7 and MDA-MB-231 breast cancer cells [24, 36]. Moreover, differential expression of GJA1 has been recently described as a potential positive prognostic marker for a clinically relevant stratification of breast cancer [23]. LM3 cells show lower levels of *Gja1* mRNA compared with SCp2 cell line (Supplementary Figure 1B). By ChIP assays we demonstrate that Runx1 is able to bind *GJA1* promoter region in MDA-MB-231 (Figure 6A). Moreover, DN/Runx1-transfected tumor cells showed a significant upregulation of *GJA1* in this cell line demonstrating an inhibitory role of Runx1 on GJA1 promoter (Figure 6B). These results strongly suggest that Runx1 is able to inhibit *GJA1* gene expression in human breast tumor cells. To show that FOXP3 is interfering with RUNX1 transcriptional inhibitory activity on GJA1 gene, we proceed to knockdown Foxp3 expression in SCp2 cells. To evaluate if Foxp3 is also able to interfere with Runx1 transcriptional inhibitory activity, we evaluated GJA1 expression in Foxp3 knockdown cells. Interestingly, reduced Foxp3 gene expression in SCp2 cell line showed a significant reduction of *Gja1* gene expression compared with transfected control cells (Figure 6C). Therefore, we propose that GJA1 is a target of Runx1 transcriptional inhibitory activity, which is repressed by Foxp3 in mammary epithelial cells.

Collectively, these observations indicate that a decreased in the availability of Foxp3 could cause a decisive increase in Runx1 transcriptional activity, which may alter migration abilities of tumor cells by binding and regulating it's downstream target genes such as *Rspo3* and *Gja1*. These data strongly suggest that Runx1/Foxp3 interaction could be a new mechanism of gene expression regulation during breast cancer development.

## DISCUSSION

In this study we show for the first time that endogenous Runx1 and Foxp3 physically interact in mammary epithelial cells leading to the inhibition of Runx1 transcriptional activity. Furthermore, we demonstrate that Runx1 is able to actively promote oncogene Rspo3 and prevent GJA1 gene expression in mammary tumor cell lines.

Although Runx1 participation has been widely described in the hematopoietic system and leukemia, during the last years different studies have indicated that this transcription factor could be also involved in other cancer types [11]. Particularly, Runx1 has been identified as a significant controller of tumorigenesis in various epithelial cancers [4, 5, 37]; however, its contribution in breast cancer development is still under debate [9]. Experimentally, Ras-mediated transformation of mammary epithelial cells (MCF10A) has been shown to be associated with loss of Runx1 expression [10]. Other report by Wang and colleagues indicated that RUNX1 knockdown causes hyperproliferation and abnormal morphogenesis in MCF10A-5E cell clone [12]. These authors also show that RUNX1 downregulation is associated with compensatory upregulation of FOXO1, since RUNX1-depleted cells require normal FOXO function for proliferation [12]. Our work shows by lost-of-function assays that Runx1 is able to modulate mammary gene expression towards a cancer behavior fate. Our data indicates that reduced Runx1 transcriptional activity downregulates *Rspo3* oncogene expression, upregulates *GJA1* tumor suppressor gene expression, significantly delays tumor cell migration and wanes cell number, in a susceptible manner to Foxp3's stalling activity. These results suggest that Runx1 could be acting as a tumor enhancer depending on Foxp3 availability. The difference observed between our study and the ones described above could be explained by different genetic background and/or because of the strategy used. While Wang and colleagues abolished RUNX1 expression in an immortalized cell line (MCF10-A-5E) [12], we reduced RUNX1 transcriptional activity in tumor cell lines (LM3 and MDA-DB-231) and upregulated its endogenous functional availability in normal epithelial cell line (SCp2) by RUNX1 overexpression or Foxp3 knockdown. It would be interesting to unravel in which steps of tumor induction and/or promotion is Runx1 and Foxp3 interplay defining cell fate.

Other authors also described this controversy analyzing RUNX1 expression by different techniques in human breast tumor samples [13, 38, 39]. Ferrari and colleagues performed the first comprehensive characterization of RUNX1 protein expression in breast cancer by immunohistochemistry using a large cohort of human samples [13]. They showed that high Runx1 protein expression correlated with poor prognosis in triple negative breast cancer patients [13]. On the other hand, large-scale sequencing studies in human breast cancer revealed frequent *RUNX1* mutations and deletions [38, 39]. This observation could be explained by the consideration that if Runx1 is used as a prognostic marker should be assessed not only by gene or protein expression levels but also by its functional activity. In addition to these considerations that might modulate Runx1 activity in the nucleus here we show new evidence that RUNX1 might be sequestered by Foxp3 blocking its activity as a transcriptional regulator. Several studies on leukemia have already shown that cell context and cellular dosage are relevant for RUNX1 activity [6, 40, 41]. In line with this and due to the multiple roles that RUNX1 displays, inducing (as for Rspo3) or repressing (as for GJA1) gene expression, probably depending on the interaction with other regulatory proteins Foxp3 expression/localization could be additionally used as a collaborative marker for prognosis [6]. The hypothesis that RUNX1 could affect different steps in tumor promotion has also been described in skin cancer where it was found that RUNX1 is important for both, tumor initiation and progression, while it had been previously shown that RUNX1 was not essential for adult tissue maintenance [42].

Here we provide evidence that Runx1 could be relevant for breast cancer cell survival and migration, supporting the idea that Runx1 could act as a tumor enhancer. This phenomenon has previously been described in ovarian [4] and skin cancer [5], among others epithelial neoplasias [3, 35]. In addition a relevant role of Runx1 in physiological hematopoietic cell migration and adhesion has been reported [43]. Interestingly, it has been shown that Runx1 depletion reduced prostate stem cell number without significant differences in apoptotic and necrotic cell death [44]. During the revision process of this manuscript Browne et al. has published experimental data that support part of our results [45].

We also demonstrate that *GJA1* mRNA levels are significantly upregulated when Runx1 transcriptional activity is reduced. The participation of GJAs in cancer has been studied *in vivo* and *in vitro* and results strongly suggest that these proteins are less expressed during tumor promotion enhancing migration and proliferation [46–49]. Moreover, Teleki and colleagues suggest that GJA1 and GJA6 could be used as potential positive and negative prognostic markers, respectively, for a clinically relevant stratification of breast cancers [23]. Furthermore, during the writing of this paper Giuliano and colleagues showed, using circulating and disseminated tumor cells from breast cancer patient-derived xenograft-bearing mice, that high GJA1 expression could be used to predict distant metastasis-free survival [50].

The results showed herein indicate that endogenous Foxp3 is able to physically interact with endogenous Runx1 in mammary epithelial cells, modulating its transcriptional activity. Ono and colleagues describe for the first time a physical interaction of Runx1/Foxp3 in normal regulatory T cells [8]. Our data show that downregulation of Foxp3 expression in mammary epithelial cells induces Runx1 transcriptional activity, which then upregulates *RSPO3* and reduces *GJA1* gene expression without increasing RUNX1 expression. Likewise, we demonstrate that upregulation of Foxp3 expression in mammary tumor cells reduces *Rspo3* gene expression. It has been proposed that FOXP3 participates in breast cancer development as a tumor suppressor factor directly bound to its oncogene and miR-146a promoter targets [15, 19]. Our *in silico* analyses revealed an absence of Foxp3 binding site in *RSPO3* and *GJA1* promoter regions. It has been shown that prognostic value of FOXP3 in breast cancer depends on localization of this protein as well as HER2 and ER expression [17]. Zuo and colleagues showed a high proportion of somatic mutations and deletions in FOXP3 gene in human breast cancer cells, which may include nuclear localization signals [15]. In agreement with our experimental data, accumulating evidence point out that FOXP3 activity is coordinated with multiple transcriptional regulators and Foxp3 localization may also depend on its molecular partners [51, 52]. Taken together these evidences suggest that Foxp3 localization could also define Runx1 activity in mammary epithelial cells [18]. Furthermore, our data strongly suggests this interaction as a new tumor suppressor mechanism used by Foxp3 to reduce breast tumor promotion.

These results strongly suggest that Runx1/Foxp3 physical interaction in mammary epithelial cells is a relevant mechanism to regulate gene expression that controls cell fate. *In vivo* experiments will allow us to test this hypothesis evaluating tumor initiation, stemness and metastatic models considering tumor microenvironment. Finally, facilitate Runx1/Foxp3 interaction could be use to improve anti-tumor strategies in breast cancer therapy.

## MATERIALS AND METHODS

### Cell lines

LM3 is a mouse mammary cancer cell line derived from a spontaneous malign adenocarcinoma from a BALB/c strain in the animal care area of Oncology Institute Angel H. Roffo (Argentina). This cell line was kindly given by Elisa Bal De Kier, Oncology Institute Angel H. Roffo. [29, 30, 53, 54]. Cells were grown in MEM (Gibco). SCp2 is a mouse mammary immortalized cell line kindly given by Dr. Mina Bissell, Lawrence Berkeley National Laboratory, Berkeley, USA [32]. Cells were routinely grown in DMEM/F12 (Gibco) supplemented with 2% FBS, 5 μg/ml insulin. MDA-MB-231 [55] (ATCC^®^ HTB- 26^™^) is a human breast cancer cell line routinely grown in RPMI (Gibco). All cell line were grown with 10% FBS (Internegocios SA), unless specified, 100 U/ml of penicillin and 100 μg/ml of streptomycin. Cells were kept at low passages and store at liquid nitrogen.

### In silico promoter analysis

We used database sites available on line (http://www.genomatix.de/ and http://www.cbrc.jp/research/db/TFSEARCH.html) to determine the presence of putative binding sites for the transcription factor Runx1 (TGTGGT of high affinity) at the promoter region of Rspo3 and Gja1 promoter region (−5000 bp up stream from +1 transcription site, Supplementary Table 1). No Foxp3 binding sites (consensus sequence: A/GTAAACAA) were found.

### Transient transfection assays and treatments

For transient transfections, 3 × 10^5^ cells were plated in medium without antibiotics and 24 h later were transfected with different vectors or siRNA as indicated: pCDNA3-DN: vector containing a dominant negative version of Runx1 (DN) subcloned from pMSCV2.2 using Bgl II and EcoR I enzymes; pCDNA3: control empty vector (C); pGL3-RORyT: a reporter plasmid containing the promoter of RORyT with putative sites for Runx1 upstream luciferase gene (Luc-R1) kindly given by V Lazarevic [56] (NIH, USA); pGL3: control vector without Runx1 binding sites (Luc-C); siRNA and Stealth RNAi^™^ siRNA Duplex oligoribonucleotide (Invitrogen) Foxp3: oligonucleotide containing a specific sequence to block Foxp3 transcription (siF); siRNA Control: oligonucleotide with scrambled sequence (siC) (sequences upon request). We used β-Gal vector as transfection control and TK-renilla vector for luciferase assays. Transfection assays were performed using Lipofectamine 2000 (Invitrogen) and OptiMEM (Life Technologies). After 48 h cells were harvested in lysis buffer. Luciferase levels were normalized to renilla expression measured with Promega luciferase/renilla kit according to the manufacturer protocol. β-Gal activity was measured by absorbance at 515 nm in spectrophotometer (BIORAD). For proliferation assay cells were harvested at 24, 48 or 72 hours after siRNA transfection. Cells were stained with bromophenol blue for viability and counted in Neubauer chamber.

Dominant negative version of Runx1 (DN) was generated introducing a stop codon at 826 cDNA sequence position included in the transactivation domain. This results in early translation termination and a short version of Runx1 behaving as a dominant negative over Runx1 during homodimerization.

For Foxp3 overexpression, LM3 cells were incubated with 0.05 μg/ul of Anisomycin (SIGMA) or vehicle (ethanol) as previously described [34].

### Immunofluorescence and confocal microscopy

SCp2 cells were fixed with 4% paraformaldehyde for 15 minutes at room temperature (RT), washed in PBS-tween 0.05% (PBS-T). Cells were permeabilized with 0.1% of Triton X-100 for 10 minutes at RT, and blocked with 2% BSA in PBS-T (blocking solution) for 1 h. After blocking, cells were incubated for 1 h at RT with anti-FOXP3 (SC-28705) or anti-Runx1 antibodies (SC-8564) in blocking solution. Cells were washed in PBS and incubated for 1 h at RT with anti goat-Cy5 (Santa Cruz) and anti rabbit-Cy3 (Santa Cruz), Dapi and phalloidin (Life Technologies) in blocking solution. After washes in PBS, coverslips were mounted with gelvatol. Images were taken at LSM510 Meta confocal microscope (Carl Zeiss).

### Co-immunoprecipitation assay

For immunoprecipitation SCp2 were washed, lysed, and pre-cleared with 20 μl A/G plus Agarose (Agarose, Santa Cruz) at 4°C for 30 minutes, and centrifuged at 1000 g for 5 minutes. Supernatant was incubated with or without 1 μl of Foxp3 primary antibody (SC-28705) and 20 μl of Agarose at 4°C. Immunoprecipitates were pelleted by centrifugation (1000 g, 5 minutes, 4°C), washed with PBS, and prepared for western blot (WB).

### Chromatin immunoprecipitation assays

LM3 and MDA-MD-231 cell lines were used for chromatin immunoprecipitation (ChIP) assays. ChIP were performed as previously described [57] using anti-RUNX1 (Abcam 23980) and anti-IgG (Abcam 46540, negative control). Quantification of ChIP was performed by real-time polymerase chain reaction (qPCR) using Stratagene Mx3000P^™^ instrument. The fold enrichment of target sequence in the immunoprecipitated compared with input fractions was calculated using the comparative Ct (the number of cycles required to reach a threshold concentration) method with the equation 2^(DCt (ref)-DCt (t))^. Each of these values were corrected by mouse GAPDH gene and referred as relative abundance over time zero. Primers sequences are available on request.

### Protein analysis

Total proteins were extracted from mammary tissue in lysis buffer supplemented with protease cocktail set I (Calbiochem). Proteins were resolved in 10 or 12% polyacrylamide gel, and transferred to a poly-vinylidine fluoride membrane. Primary antibodies: β-actin (SC-1616-R), Foxp3 (Abcam 14551), Runx1 (Abcam 61753), RSPO3 (R & D MAB41201), GAPDH (Abcam 9483), FLAG (Sigma F3165). Secondary antibodies: rabbit IgG (SC-2004), mouse IgG (SC-2005), rat IgG (SC-2006). The immune reactive protein bands were detected using chemiluminescence system (ECL_Plus system; GE) and the Fuji Film Image Reader (LAS-1000 Fuji Film). Densitometric analysis of protein levels was performed with ImageJ 1.34s software (Wayne Rasband, NIH). In each case obtained values were normalized as indicated in each experiment.

### RNA analysis

RNA was extracted from cellular lysates with Trizol reagent (Invitrogen) according to the manufacturer's instructions. For reverse transcription, 1 μg of total RNA was used. Retrotranscription was performed at 37°C for 60 minutes followed by 15 minutes at 72°C. For the quantitative real-time RT-PCR (qRT-PCR) 1 μl cDNA was used. All reactions were placed at Stratagene Mx3000P^™^ Instrument. Primers are available upon request. Reactions were run for 35 cycles under the following conditions: 15 seconds at 94°C, 20 seconds at 58°C–65°C (according to the primers), and 20 seconds at 72°C. The amplification of unique products in each reaction was verified by melting curve. Expression level of each gene was normalized to actin expression level using delta Ct method and specific primers. Means and standard deviation (SD) from at least three experiments were calculated and shown as fold changes with respect to the control. RSPO3 gene expression was analyzed by RT-PCR using MJ Research MiniCycler^™^ or Lambeth Multigene getting the same results on both machines. PCR products were visualized on 2% gel with ethidium bromide. Rspo3 expected band is 400 bp and 200 bp for Actin.

### *In vitro* wound-healing repair assays

Cells were grown in normal growth media to monolayer confluence. Scratches were performed with a tip, and cell debris was removed by washing with PBS. Width of each wounded area was measured using grids at three marked positions photographed (40x magnification) at the indicated times. Cell migration was quantified as the distance covered by cells in wound-healed surface from the marked positions. Results are expressed as mean ± SD of three independent experiments.

### Apoptosis assays

After 48 h of Lipofectamine transfection with Runx1 dominant negative (DN), control (C) expression vectors used or after 24 h incubation with 4% PFA, cells were incubated with Annexin V-FITC and Propidium Iodide (BD) for 10 minutes on ice. Apoptosis was analyzed by flow cytometer (BD FACS-Aria II). Data were processed using FlowJo software (Tree Star). Percentage of early plus late apoptotic cells was calculated as the ratio between Annexin-V FITC/PI- stained cells and total number of cells.

### Statistical analysis

Results were expressed as mean with SD as indicated in Figure legends. Differences were regarded as significant at *p* < 0.05. Statistical analyses were performed by Infostat software (Di Rienzo J.A., et al. InfoStat versión 2012. Grupo InfoStat, FCA, Universidad Nacional de Córdoba, Argentina).

## SUPPLEMENTARY MATERIAL FIGURE AND TABLE



## Abbreviations

ChIP: chromatin immunoprecipitation
DN: dominant-negative form of Runx1
EMSA: Electromobility shift assay
GJA1: Gap Junction protein Alpha 1
IP: immunoprecipitated
RSPO3: R spondin 3
siRNA: small interfering RNA
siF: siRNA for Foxp3
siC: siRNA control
SKP2: S-phase kinase-associated protein-2
