# Supplementary material for: RUNX1 and FOXP3 interplay regulates expression of breast cancer related genes
Source: Oncotarget. 2015 Dec 28;7(6):6552–65. doi: 10.18632/oncotarget.6771 (PMC4872732; doi:10.18632/oncotarget.6771)
Supplement: Supplementary file 1 [file oncotarget-07-6552-s001.pdf]

RUNX1 and FOXP3 interplay regulates expression of breast cancer related genes

Supplementary Materials

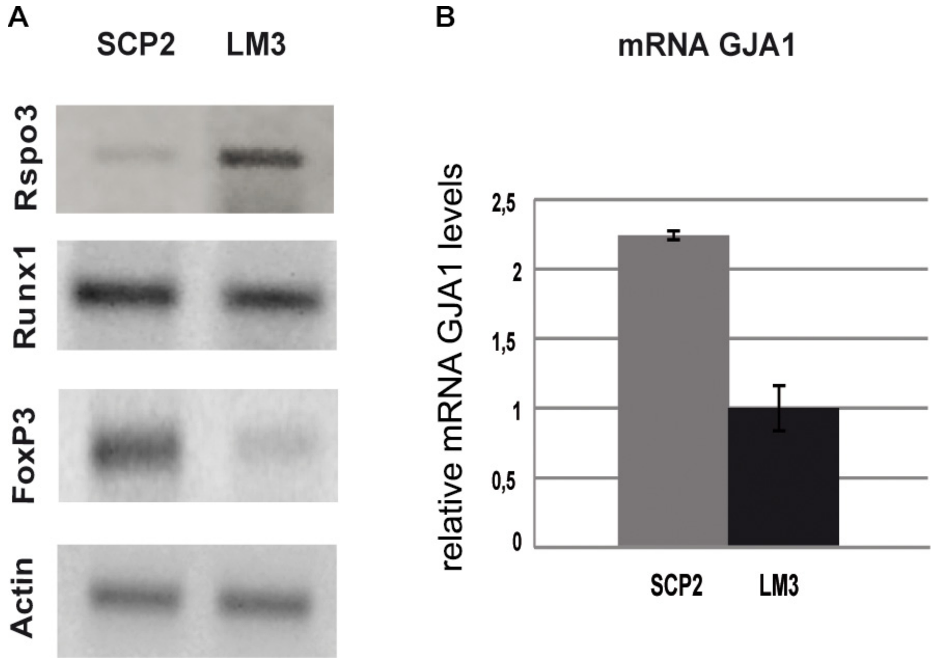

Supplementary Figure S1: Cell lines analysis. (**A**) SCp2 and LM3 cell lines were use to analyze mRNA expression of Rspo3 (first line), Runx1 (second line) and Foxp3 (third line) by RT- PCR. ACTD mRNA expression was used as loading control. (**B**) SCp2 and LM3 cell lines were use to analyze mRNA expression of Gja1 by qRT-PCR. values were normalized to *Gapdh* mRNA control.

Supplementary Table S1: Identification of Runx1 binding sites on RSPO3 and GJA1 promoter regions for human and mouse genes

Gene_Name	mRNA ID Human	Position	mRNA ID Mouse	Position
**RSPO3**	NM_032784	–1026	NM_028351.3	–2059
				–1149
				–490
				–419
**GJA1**	NM_000165	–3975	NM_010288.3	–471
				–2957
				–3059
				–3466

Runx1 high affinity binding sites positions are shown in the Table.
